# Serum BDNF as a Potential Biomarker of Alzheimer's Disease: Verification Through Assessment of Serum, Cerebrospinal Fluid, and Medial Temporal Lobe Atrophy

**DOI:** 10.3389/fneur.2021.653267

**Published:** 2021-04-23

**Authors:** Yukiko Mori, Mayumi Tsuji, Tatsunori Oguchi, Kensaku Kasuga, Atsushi Kimura, Akinori Futamura, Azusa Sugimoto, Hideyo Kasai, Takeshi Kuroda, Satoshi Yano, Sotaro Hieda, Yuji Kiuchi, Takeshi Ikeuchi, Kenjiro Ono

**Affiliations:** ^1^Department of Pharmacology, School of Medicine, Showa University, Tokyo, Japan; ^2^Pharmacological Research Center, Showa University, Tokyo, Japan; ^3^Division of Neurology, Department of Medicine, Showa University School of Medicine, Tokyo, Japan; ^4^Department of Molecular Genetics, Brain Research Institute, Niigata University, Niigata, Japan

**Keywords:** Alzheimer's disease, mild cognitive impairment, blood biomarker, BDNF, medial temporal lobe atrophy

## Abstract

There is an urgent need to establish blood biomarkers for Alzheimer's disease (AD). Although it has been speculated that brain-derived neurotrophic factor (BDNF) is associated with AD, whether it can be used as a blood biomarker has yet to be determined. We used serum, cerebrospinal fluid (CSF), and medial temporal lobe atrophy from patients with AD to evaluate the association of BDNF with AD and assess its severity. For the blood analysis, 66 participants [21 normal controls (NCs) with normal cognitive function, 22 patients with mild cognitive impairment (MCI) due to AD, and 23 patients with AD] were included. For the CSF analysis, 30 participants were included. Magnetic resonance imaging, including a voxel-based specific regional analysis system for AD, and a Mini Mental State Examination were performed. Serum levels of BDNF and CSF levels of amyloid-β_42_, total tau, and phosphorylated tau were measured using ELISA. Serum BDNF levels were significantly lower in the MCI due to AD group than in the NC group (*p* = 0.037). Although there was no significant difference in the AD group, there was a downward trend compared to the NC group. Serum BDNF levels were positively correlated with CSF Aβ_42_ levels (*r* = 0.49, *p* = 0.005). There was a significant correlation between serum BDNF levels and medial temporal lobe atrophy. Decreased serum BDNF can potentially be used as a biomarker for early AD detection. Early detection of AD with a less invasive blood test is very beneficial, as it allows for intervention before dementia progresses.

## Introduction

Alzheimer's disease (AD) is a chronic and progressive neurodegenerative disease that leads to the decline of cognitive function and gradually affects the patient's daily life. There are currently no blood biomarkers for predicting, diagnosing, or assessing the severity of AD in the way that blood glucose and HbA1c levels are used for diabetes. Establishment of such biomarkers for AD is critical for the development of disease-modifying drugs, which are expected to be the curative treatment for AD. Importantly, by the time a patient visits a medical institution with a chief complaint of cognitive decline, substantial neuronal cell death has already occurred (1). Therefore, early detection of AD and the prediction of onset risk by simple blood tests would enable therapeutic intervention before progression to neuronal cell death. This is important for providing effective treatment strategies based on biological measures that reflect the disease state of AD, thus producing a paradigm shift in the field of AD.

AD, the most common cause of dementia, is characterized by two pathological hallmarks: neurofibrillary tangles composed of hyper phosphorylated tau protein and senile plaques composed of amyloid-β protein (Aβ), which is a group of proteins composed of several fragments of different lengths. It results in synaptic loss, acetylcholine loss in the cerebral cortex, hippocampus, and fundus of the forebrain, and neuronal cell death. While the mechanisms underlying AD remain unclear, the amyloid hypothesis has sought to explain the pathophysiology of AD. This hypothesis suggests that the accumulation of Aβ aggregates, including the initial oligomers, leads to Aβ-induced lesions and an increase in neurofibrillary tangles, both of which cause neuronal cell death and, in turn, dementia (2). Importantly, the levels of Aβ_42_ in cerebrospinal fluid (CSF) are negatively correlated with the amount of cerebral Aβ deposition, and increased levels of total tau (t-tau) and phosphorylated tau (p-tau) in the CSF reflect neuronal injury (3, 4). Amyloids detected using positron emission tomography, decreased levels of Aβ_42_ in the CSF, and increased levels of t- or p-tau in CSF are the only biomarkers for detecting the pathological changes of AD. However, collection of CSF requires the use of an invasive procedure (i.e., lumbar puncture), and amyloid positron emission tomography scans are costly. Therefore, it is not possible to perform these tests on all patients with dementia in the clinical setting. Instead, diagnosis of AD is typically based on clinical symptomatology as well as imaging results. Given that blood is easier to collect with a less invasive procedure than that for CSF, the establishment of blood biomarkers specific to AD is needed. However, it has been noted that immediate applicability in clinical practice is relatively unlikely. The main limitation comes from the difficulty of measuring and standardizing thresholds among the laboratories and the potential failure to replicate the results (5).

Brain-derived neurotropic factor (BDNF) is involved in the function and survival of cholinergic neurons in the basal forebrain (6, 7). Several studies have reported that BDNF likely plays an important role in the pathological condition of AD (8–11). Therefore, BDNF has attracted attention as one of the biomarker candidates for AD and has been widely investigated. Indeed, blood BDNF levels decrease in patients with AD or mild cognitive impairment (MCI) (12–15). However, some studies have shown inconsistent findings of circulating BDNF levels in AD patients (16–18) due to methodological biases (19), thereby questioning its use as a blood biomarker. Moreover, few studies have surveyed the relationship between serum BDNF and CSF Aβ_42_ or CSF tau levels systematically. Therefore, we examined whether serum BDNF could be a useful blood biomarker for AD using serum, CSF, and imaging in patients with MCI due to AD and AD.

## Methods

### Subjects

We first analyzed blood in the participants. For blood analysis, we enrolled 66 participants, including 21 normal controls (NCs) with normal cognitive function, 22 patients with MCI due to AD, and 23 patients with AD. The participants visited or were admitted to the Department of Neurology, Showa University from June 2016 to February 2020 and agreed to have blood collected. The NC group included elderly persons who visited our department for cognitive function screening or patients who visited our department for a disease that did not affect cognitive function; all of them had normal cognitive function. Their normal cognitive function was confirmed by one of the following methods: (1) Clinical Dementia Rating Scale was normal; (2) Mini Mental State Examination (MMSE) score was ≧ 24; or (3) Detailed psychological tests, including the Wechsler Adult Intelligence Scale-Third Edition and Wechsler Memory Scale-Revised, were normal. Second, we analyzed CSF in the participants. Of the 66 participants whose blood was collected, 30 agreed to simultaneous CSF sampling. Thus, for CSF analysis, a total of 30 participants (eight NCs, 12 patients with MCI due to AD, and 10 patients with AD) were included.

Patients with AD and MCI due to AD were diagnosed based on the diagnostic criteria (3, 20) of the National Institute on Aging-Alzheimer's Association. For diagnostic purposes, magnetic resonance imaging (MRI) was performed, including the use of a voxel-based specific regional analysis system for AD (VSRAD), which is used to assess the atrophy of the medial temporal lobe. Participants who could not undergo MRI for special reasons underwent computed tomography to distinguish dementia other than AD. Consequently, we defined the following two groups as “MCI due to AD” in this study: (1) MCI due to AD-intermediate likelihood, which included those having a positive structural MRI for AD but not CSF data; and (2) MCI due to AD-high likelihood, which included those having both positive findings for CSF Aβ_42_ and a positive structural MRI. Cerebral blood flow scintigraphy was also performed. The MMSE was used to evaluate cognitive function. Apolipoprotein E (ApoE) genotype measurement was outsourced (BML, Inc., Tokyo, Japan). The following characteristics of the participants were investigated: educational level, body mass index, smoking habit, alcohol consumption, medications (benzodiazepine or non-benzodiazepines, lipid-lowering drugs, anticoagulant or antiplatelet drugs, antihypertensives, oral diabetic drugs) (Tables 1, 2). All participants were given written explanations regarding the study, and they provided written consent. This study adhered to the tenets of the Declaration of Helsinki and was approved by the ethics committee of Showa University.

**Table 1:** Characteristics of participants included in the serum analysis.

	**NC (*n* = 21)**	**MCI due to AD (*n* = 22)**	**AD (*n* = 23)**	*p*-value^*^
Sex (M/F)	8/13	13/9	6/17	0.075
Age (years)	74.3 ± 9.4	78.6 ± 7.7	75.4 ± 10.8	0.234
Education level (years)	13.4 ± 2.3	14.0 ± 2.9	12.6 ± 2.7	0.521
BMI (kg/m^2^)	22.3 ± 3.6	23.9 ± 4.2	20.2 ± 2.5‡	0.005
Smoking habit				0.084
Never smoked	11	15	16	
Former smoker	5	4	2	
Current smoker	3	0	0	
Alcohol consumption				0.961
Abstainer	15	12	13	
A few times in a week	4	4	4	
Medications				
Benzodiazepines/Non-benzodiazepines	5	6	4	0.724
Lipid-lowering drugs	7	13	9	0.199
Anticoagulant/antiplatelet drugs	3	8	3	0.103
Antihypertensives	9	15	11	0.207
Oral diabetic drugs	4	4	4	0.990
MMSE score	27.6 ± 1.6	24.6 ± 2.9^†^	17.3 ± 4.6‡§	<0.0001
ApoE genotype				0.023
ε2/ε3	1	0	0	
ε3/ε3	14	4	10	
ε3/ε4	1	8	8	
ε4/ε4	0	2	2	
BDNF (ng/ml)	35.7 ± 13.4	28.9 ± 13.6^†^	31.4 ± 11.8	0.037
VSRAD				
Severity	0.97 ± 0.52	2.18 ± 1.05^†^	2.12 ± 1.14§	0.001
Extent (%)	8.37 ± 16.7	43.2 ± 29.5^†^	38.5 ± 31.7§	0.001
Whole GM (%)	3.42 ± 1.80	6.50 ± 3.32^†^	7.12 ± 2.84§	0.001
Ratio	2.49 ± 4.18	6.76 ± 4.35^†^	5.57 ± 4.91	0.011

*Data are presented as means ± standard deviations or absolute numbers.*

*NC, normal control; MCI, mild cognitive impairment; AD, Alzheimer's disease; BMI, body mass index; MMSE, Mini Mental State Examination; ApoE, apolipoprotein E; BDNF, brain-derived neurotrophic factor; VSRAD, voxel-based specific regional analysis system for Alzheimer's disease; GM, gray matter.*

* *Kruskal–Wallis test. p values among the three groups are indicated in the table. §Bonferroni test: Significant differences in NC vs. AD were observed in the MMSE score (p < 0.0001), VSRAD Severity (p = 0.003), Extent (p = 0.004), and Whole GM (p = 0.001).*

† *Significant differences in NC vs. MCI due to AD were observed in the MMSE score (p = 0.043), BDNF (p = 0.037), VSRAD Severity (p = 0.001), Extent (p = 0.001), Whole GM (p = 0.023), and Ratio (p = 0.009). ‡Significant differences in MCI due to AD vs. AD were observed in the BMI (p = 0.004) and MMSE score (p < 0.0001)*.

**Table 2:** Characteristics of participants included in the CSF analysis.

	**NC (*n* = 8)**	**MCI due to AD (*n* = 12)**	**AD (*n* = 10)**	*p*-value^*^
Sex (M/F)	5/3	8/4	3/7	0.191
Age (years)	67.4 ± 8.8	76.7 ± 8.6	71.8 ± 13.1	0.159
MMSE score	27.7 ± 2.1	24.5 ± 3.1	16.8 ± 5.5^‡^^§^	0.002
Aβ_42_ (pg/ml)	443.4 ± 223.4	254.2 ± 76.9	202.7 ± 62.2^§^	0.002
t-tau (pg/ml)	283.2 ± 151.0	422.4 ± 275.5	428.4 ± 145.2	0.226
p-tau (pg/ml)	54.4 ± 16.8	73.9 ± 33.8	69.0 ± 21.7	0.296

*Data are presented as means ± standard deviations.*

*CSF, cerebrospinal fluid; NC, normal control; MCI, mild cognitive impairment; AD, Alzheimer's disease; MMSE, Mini Mental State Examination; Aβ, amyloid-β protein; t-tau, total tau; p-tau, phosphorylated tau.*

* *Kruskal–Wallis test: p-values among the three groups are indicated in the table.*

‡ *Bonferroni test: A significant difference in MCI due to AD vs. AD was observed in the MMSE score (p = 0.014).*

§ *Significant differences in NC vs. AD were observed in the MMSE score (p = 0.007) and Aβ_42_ levels (p = 0.001)*.

### Serum and Cerebrospinal Fluid Sampling

Serum samples were obtained between 09:00 h and 16:00 h. CSF samples were obtained simultaneously. The collected serum and CSF samples were immediately separated by centrifugation. Serum was centrifuged at room temperature for 10 min at 1,300 × *g*, and CSF was centrifuged at room temperature for 10 min at 400 × *g*. They were frozen at −80°C until analysis. At the time of the immunoassay, they were gently thawed on ice.

### Serum Measurement

Serum BDNF levels were measured using an ELISA. The measurement kit used was the Mature BDNF Rapid^TM^ ELISA Kit: Human, Mouse, Rat (No. BEK-2211-1P/ 2P; Biosensis Pty. Ltd., Thebarton, Australia). All measurement procedures were performed according to the manufacturer's instructions.

### Cerebrospinal Fluid Measurement

The concentrations of Aβ_42_ and t- and p-tau were analyzed at the Department of Molecular Genetics, Brain Research Institute, Niigata University, Niigata, Japan. Aβ_42_ was measured using V-PLEX Aβ Peptide Panel 1 (6E10) (Meso Scale Discovery, Rockville, MD, USA) with MESO QuickPlex SQ 120 (Meso Scale Diagnostics, LLC, Rockville, MD, USA). T- and p-tau were measured using the commercially available ELISA kit, INNOTEST hTAU Ag and PHOSPHO-TAU (181P) (Fujirebio Europe, Inc., Ghent, Belgium). The laboratory at Niigata University participates in the Alzheimer's Association external quality control program for CSF biomarkers.

### Medial Temporal Lobe Atrophy Assessment

Analysis of MRI images was performed using the VSRAD software (VSRAD® Advance 2, Eisai Co., Ltd., Kamisu, Japan) to determine the extent of gray and white matter atrophy (21). VSRAD software is widely used in the clinical diagnosis of AD in Japan. It quantitatively calculates the extent of brain atrophy (percent of volume reduction in gray and white matter) compared to an MRI database of 80 age-matched healthy controls based on voxel-based morphometry. Z-scores [(NC average of voxel-level-patient's voxel-level)/(NC standard deviation)] were calculated in each voxel, and the areas with a Z-score ≥2 were considered atrophied. In the clinical diagnosis of AD, medial temporal lobe atrophy is a pivotal feature. Therefore, this area was targeted as the volume of interest (VOI). We used four VSRAD scores for imaging analysis in this study (22): (1) A Z-score of gray matter (GM) atrophy severity in the VOI (“Severity”); (2) the extent of GM atrophy in the VOI (“Extent”); (3) the extent of GM atrophy in the whole brain (“Whole GM”); and (4) the ratio of the extent of GM atrophy in the VOI to that in the whole brain (“Ratio”). The two indicators, “Extent” and “Whole GM,” are expressed as the percentage of the area with a Z-score ≥2. The fourth indicator, “Ratio,” shows the strength of selective atrophy.

### Statistical Analysis

Data are presented as means ± standard deviations. The differences between the two groups were assessed using the Mann–Whitney U test. The differences among the three groups were assessed using the Kruskal–Wallis test followed by a Bonferroni/Dunn test for multiple comparisons or the chi-square test. The relationship between variables was ascertained using Spearman's rank correlation coefficient. The results were considered significant when *p*-value was < 0.05. All statistical analyses were performed using IBM SPSS Statistics for Windows, version 26.0 (IBM Corp., Armonk, NY, USA).

## Results

The results of serum and CSF measurements are shown in Tables 1, 2, respectively.

**Figure 1:**
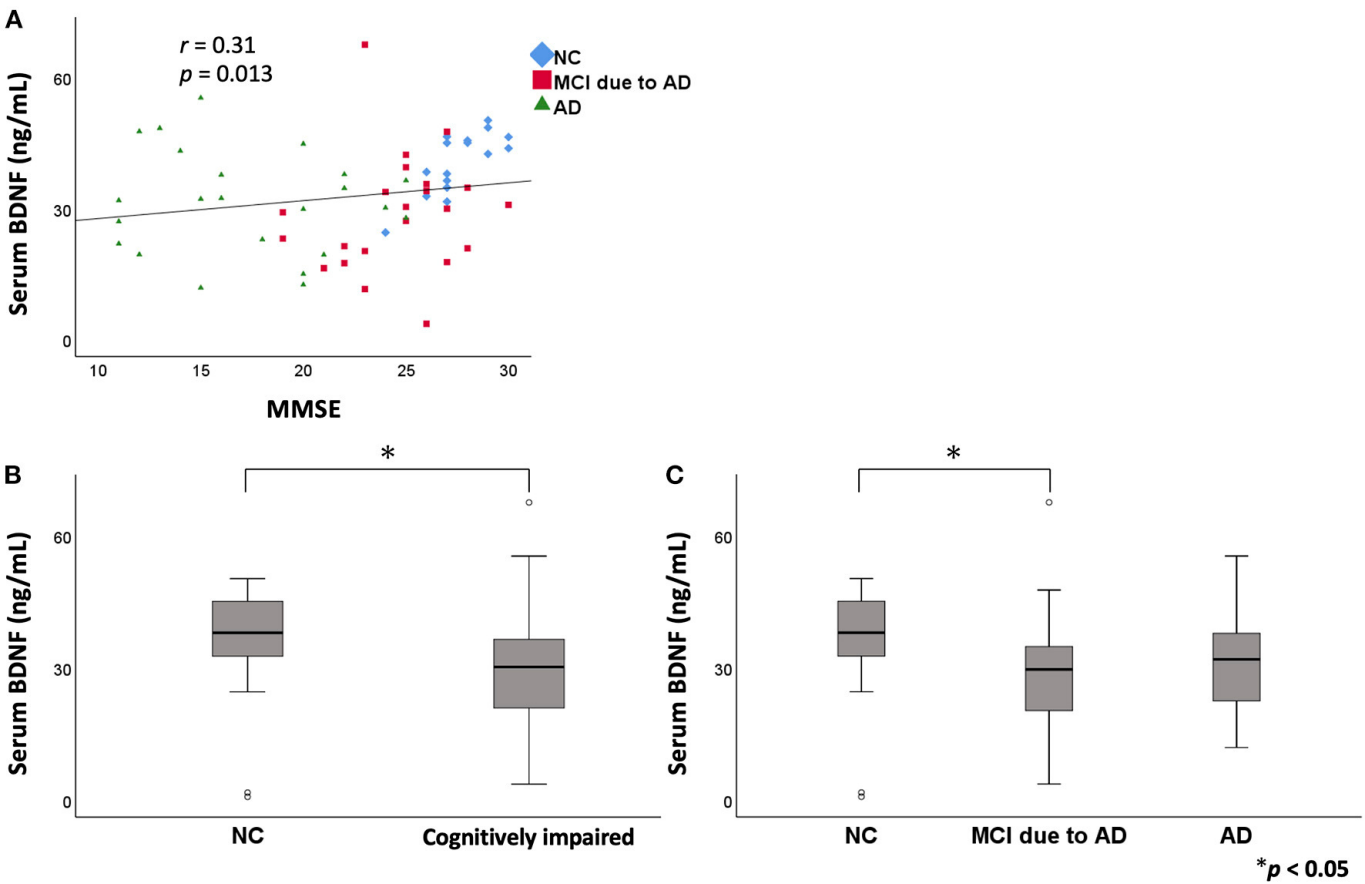
The relation between serum BDNF levels and MMSE. Comparison of serum BDNF levels among NC, MCI due to AD, and AD groups. A positive correlation was found between serum BDNF levels and MMSE score (*r* = 0.31, *p* = 0.013) **(A)**. On comparing serum BDNF levels between the NC group and cognitively impaired group (the MCI due to AD and AD groups), the cognitively impaired group had significantly lower levels than those of the NC group (*p* = 0.015) **(B)**. BDNF serum levels were significantly different among the three groups (*p* = 0.037). The MCI due to AD group had significantly lower serum levels compared to those of the NC group (*p* = 0.037) **(C)**. BDNF, brain-derived neurotrophic factor; MMSE, Mini Mental State Examination; NC, normal control; MCI, mild cognitive impairment; AD, Alzheimer's disease.

In the serum analysis, there was no significant difference in age among the three groups [NC (mean ± standard deviation): 74.3 ± 9.4 years, MCI due to AD: 78.6 ± 7.7 years, AD: 75.4 ± 10.8 years]. MMSE score was significantly lower in the MCI due to AD and AD groups compared to the NC group. A positive correlation was found between serum BDNF level and MMSE score (*r* = 0.31, *p* = 0.013; Figure 1). Comparing serum BDNF levels between the NC group and cognitively impaired group (the MCI due to AD and AD groups), the cognitively impaired group had significantly lower BDNF levels than that in the NC group (*p* = 0.015). BDNF serum levels were significantly different among the three groups (*p* = 0.037). The MCI due to AD group had significantly lower serum levels compared to those of the NC group (*p* = 0.037; Figure 1). Although there was no significant difference in the AD group, a downward trend compared to the NC group was observed. In the three groups, serum BDNF levels did not correlate with age (NC: *r* = - 0.005, *p* = 0.982; MCI due to AD: r = −0.16, *p* = 0.460; AD: *r* = 0.31, *p* = 0.140). In the three groups, serum BDNF levels did not show any significant differences with respect to sex (NC, *p* = 0.916; MCI due to AD, *p* = 1.000; AD, *p* = 0.319).

**Figure 2:**
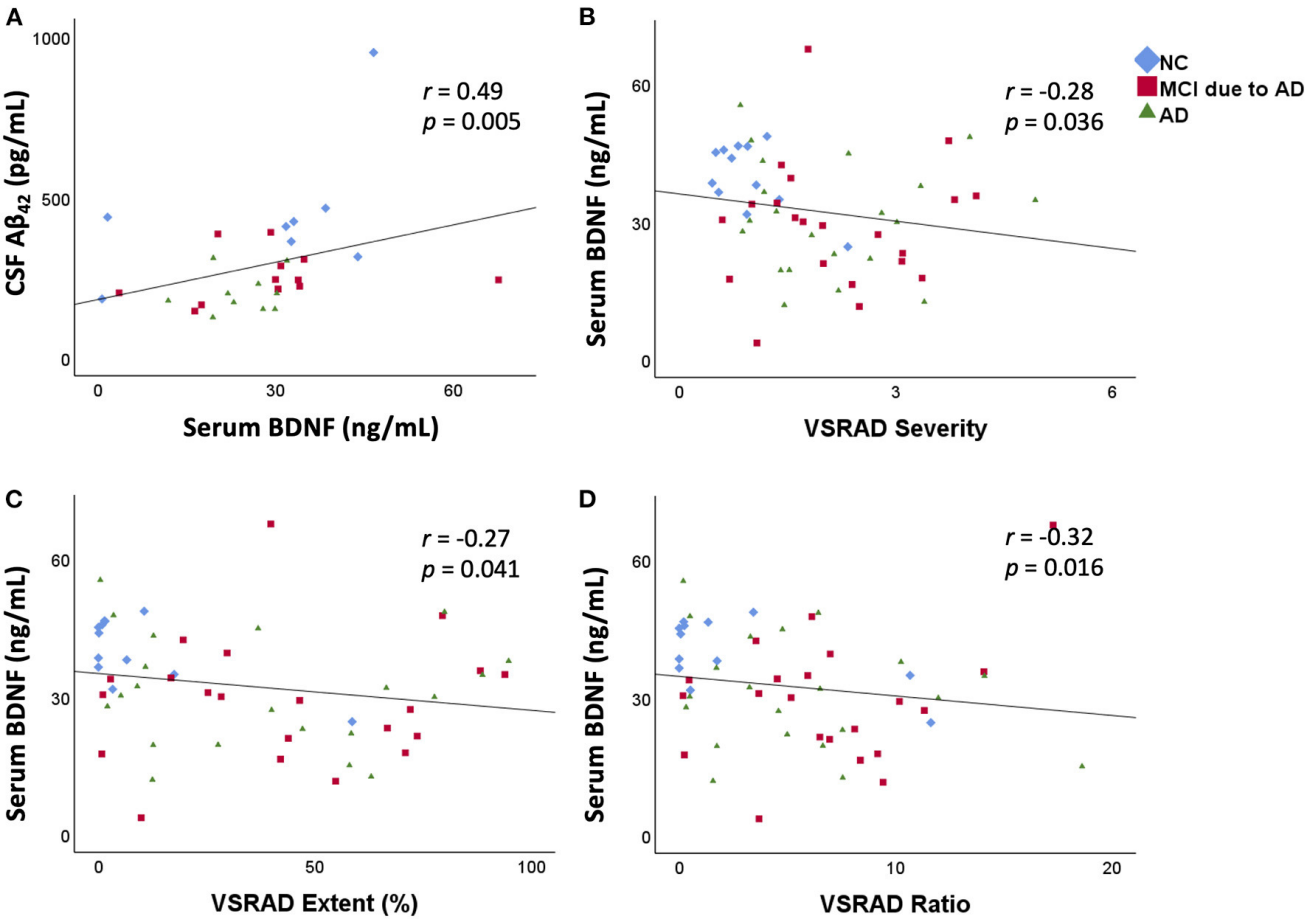
Correlation between serum BDNF levels and CSF Aβ_42_ levels and the relation between serum BDNF levels and medial temporal lobe atrophy. Serum BDNF levels were positively correlated with CSF Aβ_42_ levels (*r* = 0.49, *p* = 0.005) (**A**). Serum BDNF levels were significantly negatively correlated with VSRAD Severity (**B**), VSRAD Extent (**C**), and VSRAD Ratio (**D**). VSRAD software, for the analysis of MR images, is widely used in the clinical diagnosis of AD in Japan for assessment of medial temporal lobe atrophy. In the present study, we used four VSRAD scores for imaging analysis (22). VSRAD Severity indicates the degree of atrophy of the medial temporal lobe region associated with memory. VSRAD Extent is the ratio of the atrophic area in the medial temporal lobe region. VSRAD Ratio shows the strength of selective atrophy. BDNF, brain-derived neurotrophic factor; CSF, cerebrospinal fluid; Aβ, amyloid-β protein; AD, Alzheimer's disease; NC, normal control; MCI, mild cognitive impairment; VSRAD, voxel-based specific regional analysis system for AD.

In the CSF analysis, there was no significant difference in age among the three groups [NC (mean ± standard deviation): 67.4 ± 8.8 years, MCI due to AD: 76.7 ± 8.6 years, AD: 71.8 ± 13.1 years]. Serum BDNF levels were positively correlated with CSF Aβ_42_ levels (*r* = 0.49, *p* = 0.005; Figure 2). There was no correlation between serum BDNF levels and either CSF t-tau or p-tau levels.

The results of medial temporal lobe atrophy are shown in the lower part of Table 1. The scores of “Severity,” “Extent,” and “Whole GM” of VSRAD were significantly higher in the MCI due to AD and AD groups than in the NC group. The “Ratio” of VSRAD was significantly higher in the MCI due to AD group than that in the NC group. A negative correlation was found between serum BDNF levels and the VSRAD “Severity” (*r* = −0.28, *p* = 0.036), “Extent” (*r* = −0.27, *p* = 0.041), and “Ratio” (*r* = −0.32, *p* = 0.016) (Figure 2).

## Discussion

In the present study, we demonstrated that the serum BDNF levels in the MCI due to AD group were significantly lower than those in the NC group. Furthermore, a positive correlation was found between serum BDNF levels and CSF Aβ_42_ levels, which is one of the few biomarkers for AD. Moreover, to date, this association has not been previously demonstrated. Establishing blood biomarkers remains challenging due to the difficulty in demonstrating their association with CSF Aβ and tau, the only known biomarkers of AD. Our study may support the potential of serum BDNF as a blood biomarker for AD.

BDNF is a neurotrophic factor that controls the differentiation and growth of neural progenitor cells and promotes neurite outgrowth. BDNF production in the brain controls synapse formation and pruning, as well as neural plasticity associated with learning and memory, and maintains normal brain function. Although the association between cognitive decline and reduction in blood BDNF levels has been reported, it has also been shown to decrease in AD (12, 13) and MCI (14, 15). There is no consensus regarding the relationship between the severity of AD and serum BDNF levels (19). In the present study, we found that although there was no significant difference in the AD group, the MCI due to AD group had significantly lower serum BDNF levels compared to the NC group. This indicates that a decrease in serum BDNF may occur during the early stages of AD pathogenesis, i.e., Aβ accumulation (23), probably based on recently reported molecular mechanisms of Aβ-induced decrease of BDNF (24, 25).

Given that BDNF crosses the blood–brain barrier (26) and the main source of circulating BDNF is believed to be the brain (27), decreased serum BDNF levels may reflect decreased BDNF levels in the CSF. We tried to estimate BDNF levels in the CSF, but it was difficult to measure by the kit for serum BDNF detection. Therefore, it was not possible to demonstrate a decrease in CSF BDNF levels parallel to a decrease in serum BDNF levels in this study.

It has been demonstrated that exercise stimulates the expression of BDNF in the hippocampus (28), increases hippocampal volume, and improves memory (29). Therefore, changes in serum BDNF levels may also be due to the individual amount of physical activity. Apathy, which is manifested by cognitive decline, may lead to a reduction in exercise and contribute to lower serum BDNF levels. However, in this study, the participants' exercise habits and amount of exercise were not considered. This may be the underlying reason for no significant difference in BDNF levels between the NC and AD groups in this study.

A correlation was found between medial temporal lobe atrophy and decreased serum BDNF levels in the present study. Medial temporal lobe atrophy was observed in the MCI due to AD and AD groups compared to the NC group. The association between serum BDNF levels and medial temporal lobe atrophy has been investigated in relation to an age-related decrease in hippocampal volume (30) and severity of AD (31). To the best of our knowledge, the present study is the first to show a correlation between serum BDNF levels and medial temporal atrophy in relation to the severity of AD (Figure 2). A decrease in serum BDNF levels was associated with a decrease in CSF Aβ_42_ levels, but the mechanism for this could not be determined based on the previous findings. Decreased CSF Aβ_42_ level means Aβ aggregation in the central nervous system. Aβ aggregation is known to trigger the progression of tau pathology (32). It is thought that the progression of tau pathology causes neuronal loss and medial temporal lobe atrophy, which may explain the correlation between decreased serum BDNF levels and medial temporal lobe atrophy in this study. However, we could not show the correlation between serum BDNF levels and either CSF t-tau or p-tau levels. The underlying reason for no correlation between serum BDNF levels and CSF tau levels may be because neither MCI due to AD nor AD patients showed a significant increase in CSF tau levels. It may also indicate that BDNF levels are more closely associated with Aβ levels than tau levels. Aβ is a marker that shows changes from the early stage of AD compared to tau (23). Patients with decreased serum BDNF levels may have decreased CSF Aβ_42_ levels and medial temporal lobe atrophy, which may also be useful as diagnostic biomarkers for AD.

Our results show that serum BDNF, for which consensus has not yet been reached, may be used as a biomarker for diagnosing early AD in clinical practice because it may reflect Aβ aggregation in the brain. Findings at the MCI due to AD stage are essential in treating dementia, since they may be helpful in preventing progression of dementia to AD. Early detection of AD with a less invasive blood test is very beneficial, as it facilitates early intervention. Furthermore, it may allow those who are conscious of cognitive decline to know their MCI status. This study has a few limitations. First, the sample size was small, and the lifestyle habits such as the participants' exercise habits were not considered. Second, the analyses were based on cross-sectional data. Future studies with large cohorts are required, and longitudinal studies are warranted to test the potential of serum BDNF as a prognostic tool for AD.

## Data Availability Statement

The raw data supporting the conclusions of this article will be made available by the authors, without undue reservation.

## Ethics Statement

The studies involving human participants were reviewed and approved by the ethics committee of Showa University. The patients/participants provided their written informed consent to participate in this study.

## Author Contributions

YM, MT, and KO contributed to the conception and design of this research. YM, TO, SH, MT, and KO contributed to the analysis and interpretation of the data as well as the initial drafting of the manuscript. YM, TO, and KK performed all the experiments. All authors contributed to the acquisition of the data, critically revised it for important intellectual content, and approved its final version and are accountable for the contents of this work.

## Conflict of Interest

The authors declare that the research was conducted in the absence of any commercial or financial relationships that could be construed as a potential conflict of interest.
